# Modular Pulse Program Generation for NMR Supersequences

**DOI:** 10.1021/acs.analchem.1c04964

**Published:** 2022-01-20

**Authors:** Jonathan R. J. Yong, E̅riks Kupče, Tim D. W. Claridge

**Affiliations:** † Chemistry Research Laboratory, Department of Chemistry, University of Oxford, Mansfield Road, Oxford OX1 3TA, United Kingdom; ‡ Bruker UK Ltd, R&D, Coventry CV4 9GH, United Kingdom

## Abstract

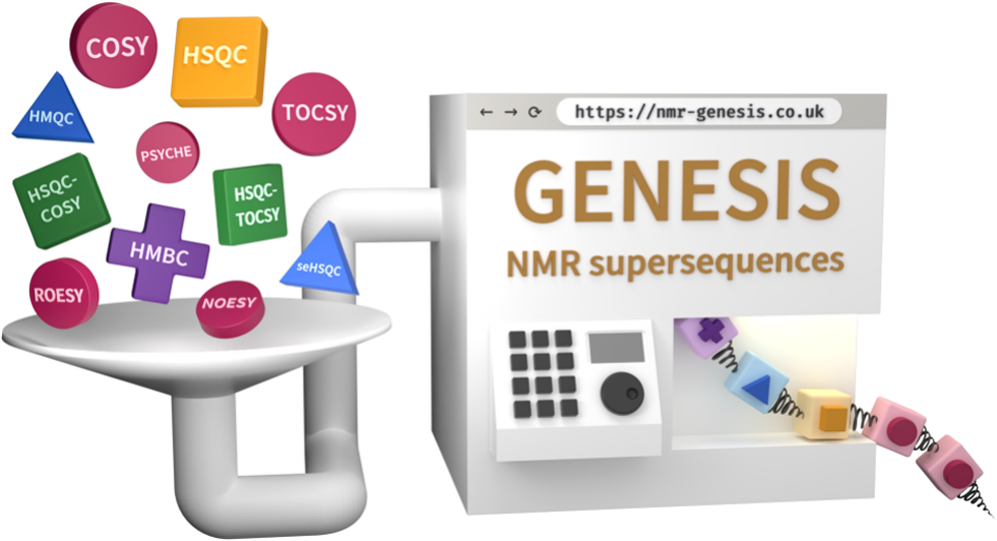

NMR supersequences allow multiple 2D NMR data sets to be acquired in greatly reduced experiment durations through tailored detection of NMR responses within concatenated modules. In NOAH (NMR by Ordered Acquisition using ^1^H detection) experiments, up to five modules can be combined (or even more when parallel modules are employed), which in theory leads to thousands of plausible supersequences. However, constructing a pulse program for a supersequence is highly time-consuming, requires specialized knowledge, and is error-prone due to its complexity; this has prevented the true potential of the NOAH concept from being fully realized. We introduce here an online tool named GENESIS (GENEration of Supersequences In Silico), available via https://nmr-genesis.co.uk, which systematically generates pulse programs for arbitrary NOAH supersequences compatible with Bruker spectrometers. The GENESIS website provides a unified “one-stop” interface where users may obtain customized supersequences for specific applications, together with all associated acquisition and processing scripts, as well as detailed instructions for running NOAH experiments. Furthermore, it enables the rapid dissemination of new developments in NOAH sequences, such as new modules or improvements to existing modules. Here, we present several such enhancements, including options for solvent suppression, new modules based on pure shift NMR, and improved artifact reduction in HMBC and HMQC modules.

NMR spectroscopy is one of the most important analytical techniques for the characterization of molecular structures. In particular, *n*-dimensional (*n*D) NMR experiments (*n* ≥ 2) provide extensive information about through-bond and through-space connectivity. However, such experiments require the incrementation of one or more indirect-dimension evolution periods, leading to long experiment times. The acceleration of *n*D NMR has therefore emerged as a highly popular area of research: developments in this area include (but are not limited to) ultrafast NMR,^1−4^ nonuniform sampling (NUS),^5−7^ multiple-FID experiments,^4,8−11^ and the shortening or elision of recovery delays.^12−15^ NOAH (NMR by Ordered Acquisition using ^1^H detection) experiments,^4,16−24^ which encompass the last two categories, consist of a series of multiple 2D experiments (“modules”), combined into one single “supersequence” that uses only one recovery delay for all modules. This provides up to 4× time savings compared to conventional acquisition, in which one recovery delay is used per module (Figure 1).

**Figure 1:**
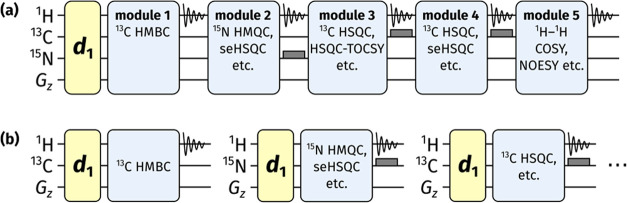
(a) Diagrammatic representation of a NOAH supersequence, which consists of up to five modules but uses only one recovery delay (*d*_1_). Filled gray bars indicate heteronuclear decoupling during acquisition periods. (b) Conventional 2D NMR data acquisition, where one recovery delay is used per module.

Virtually all common 2D experiments employed for small-molecule characterization have been implemented in NOAH supersequences to date, including HMBC, HSQC, HSQC-TOCSY, HMQC, COSY, TOCSY, NOESY, and ROESY. Each module is given a unique abbreviation, usually one letter long (e.g., “B” for HMBC, “S” for HSQC, “M” for HMQC, “C” for COSY) and occasionally sub/superscripted (e.g., “S^T^” for HSQC-TOCSY). The combinatorial nature of NOAH experiments means that there are a very large number of conceivable supersequences ranging from NOAH-2 to NOAH-5 (where the suffix indicates the number of modules); the use of parallel “*p*-NOAH” supersequences^24^ extends this maximum number even further.

For optimal data quality in terms of both sensitivity and artifact minimization, there are certain restrictions on NOAH supersequences. Specifically, NOAH modules placed earlier in a supersequence should ideally only excite the magnetization they need, leaving all other magnetization sources untouched; provided this is obeyed, the resulting NOAH spectra effectively have sensitivity comparable to conventional experiments. As an example, in the NOAH-2 SC supersequence (comprising HSQC and COSY modules), the ^1^H–^13^C HSQC module is designed to excite only the ^1^H nuclei directly attached to the 1.1%-natural abundance ^13^C and leave all other proton magnetization (the “bulk magnetization”) in the equilibrium state, i.e., along the +*z*-axis.^14^ A ^1^H–^1^H COSY module (or TOCSY, or NOESY, etc.) can then draw on this bulk magnetization, with almost no loss in sensitivity and without having to wait for the ^12^C-bound protons to relax. Conversely, if the COSY module were placed first, the ^13^C-bound proton magnetization would not survive for use in the HSQC: thus, the HSQC module in a NOAH-2 CS would display severe sensitivity losses compared to a NOAH-2 SC. More subtle factors also apply, such as in the SBC sequence,^16^ where COSY intensities are modulated by *T*_2_ relaxation and *J*_HH_ evolution. The alternative BSC arrangement,^17^ especially with isotropic “ASAP” mixing applied before the COSY module, circumvents this issue and has become the preferred implementation for HMBC/HSQC combinations.^18^ Although the HMBC and COSY modules in this supersequence excite the same magnetization pool, the resulting sensitivity losses in the COSY spectrum are readily tolerated as it has a far greater intrinsic sensitivity compared to the HMBC spectrum.

Considerations such as these restrict the number of “viable” NOAH supersequences, where time savings are not undermined by detrimental sensitivity losses. Even so, the original NOAH paper alone suggests a figure of 285,^16^ and the number of available modules has only grown since then. By our calculations, as of the time of writing, there are 4242 viable NOAH supersequences (Section S1, Supporting Information). Although there is a clear blueprint for how to construct such supersequences, it is evidently impractical to do all of these “by hand.” Each combination requires its own pulse program, which is typically many hundreds of lines long; this complexity makes pulse program construction highly time-intensive and makes the chances of errors (such as inconsistent parameter definitions) substantially higher. Consequently, of the thousands of combinations available, only a few dozen “typical” supersequences have been released, representing only a fraction of the possibilities which the NOAH technique offers; these alone are unlikely to adequately meet the varied requirements of users.

To solve this problem, we sought to *programmatically* generate NOAH pulse programs, an approach which we term GENESIS (GENEration of Supersequences In Silico). A programmatic approach not only provides virtually instantaneous results but also ensures that the output is predictable and can be reasoned about, which eliminates many possibilities for user error during pulse program construction. The modular nature of NOAH supersequences (i.e., having multiple almost-independent components which are pieced together to form a greater entity) lends itself well to this, as each component only needs to be defined once to be usable in all combinations. GENESIS is implemented in the form of a single web page (Figure 2), accessible via https://nmr-genesis.co.uk, which can construct virtually any supersequence one might want and output a Bruker pulse program ready for download and execution. Apart from allowing users to download customized supersequences, this allows new NOAH developments to be easily and rapidly disseminated to users, independently of Bruker’s own release cycle and without requiring a separate publication for each. Some such enhancements (namely, solvent suppression options, new pure shift-based modules, improved versions of HMBC and HMQC modules which minimize spectral artifacts, and various streamlined aspects of acquisition and processing) are detailed later in this article; we anticipate that future feature requests from the NMR community will be similarly implemented.

**Figure 2:**
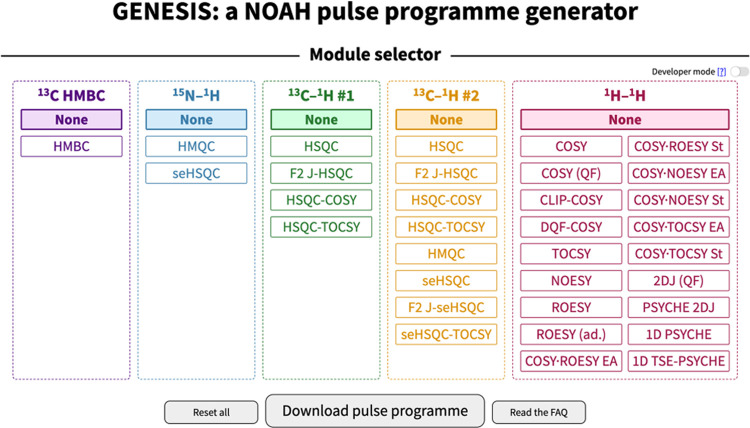
Screenshot of the GENESIS web interface. Visible here are the module choices, the “developer mode” toggle, and the button for downloading the pulse program.

The regular GENESIS user interface is designed to produce only viable supersequences: thus, for example, it is not possible to create the CS or SBC supersequences discussed earlier. This is most useful for users who wish to follow established best practices. For more advanced usage, enabling developer mode will remove these limitations, allowing any arbitrary combination of modules to be created. The website further contains an extensive library of frequently asked questions about the implementation and practical details of NOAH experiments. It also offers download links for the AU scripts used for processing, as well as a new Python script used for toggling nonuniform sampling on or off.

## Implementation Details

We begin with a brief discussion of how the GENESIS approach works. The pulse program generation code itself is written in TypeScript (version 4.2.3, Microsoft), which is compiled to JavaScript (formally ECMAScript 2015, or “ES6”) and then executed directly in a client’s web browser. The interface displays a list of modules for users to choose from, using accessible and familiar names such as HMBC, HSQC, and so on (Figure 2). Internally, these are mapped to a series of objects, each of which contain module-specific information, such as its abbreviation, the requisite parameter definitions, the pulse program for the module itself, and the appropriate AU program to be used for processing.

**Figure 3:**
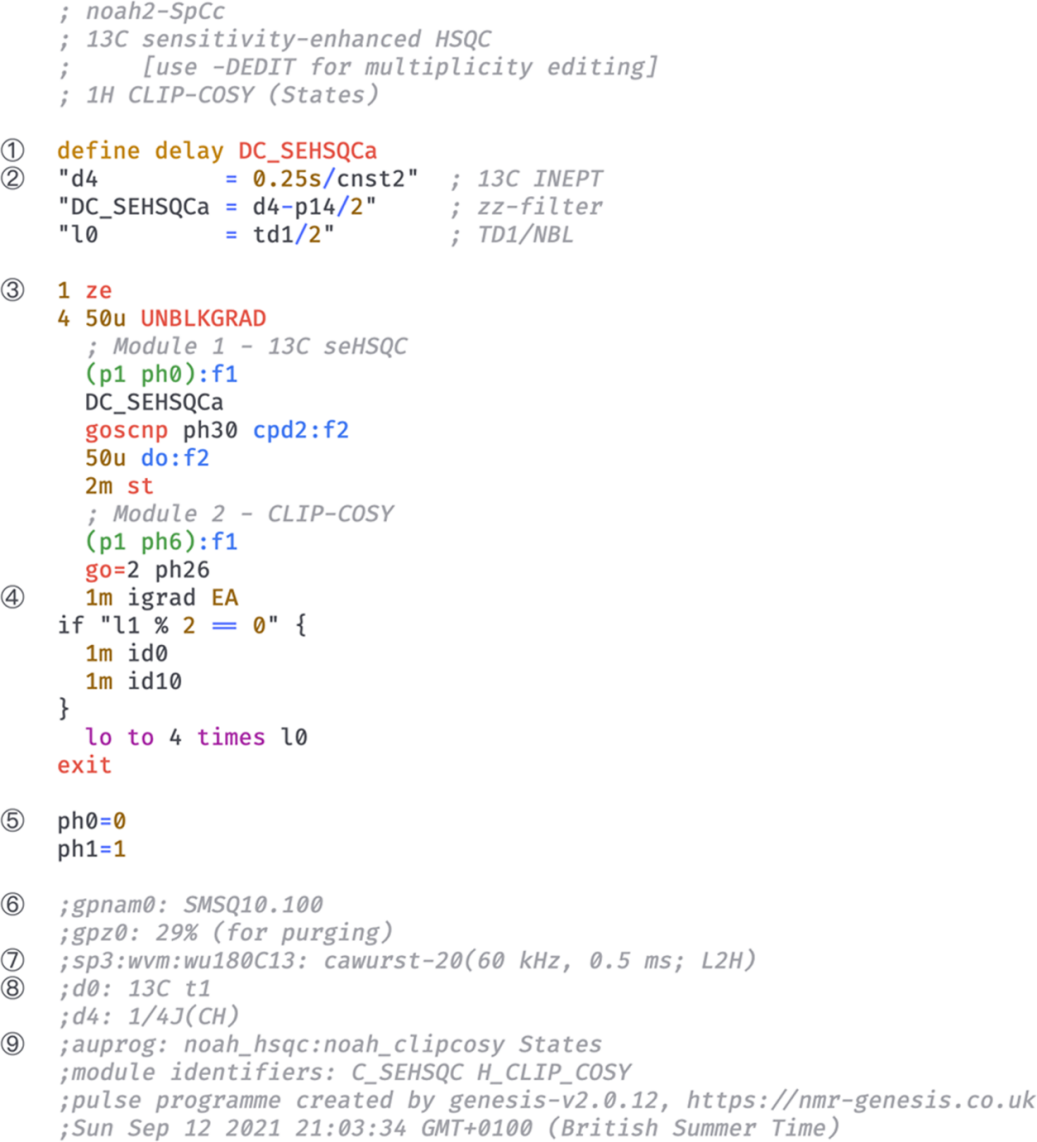
Abridged GENESIS pulse program for a NOAH-2 S^+^C^c^ supersequence (^13^C seHSQC + CLIP-COSY). Specific sections of interest are numbered on the left. (1) Module-specific delays are given unique identifiers to prevent clashes and to improve readability. (2) TopSpin parameters (such as the delay *d4*) are standardized between modules. (3) The pulse program instructions begin here. (4) Commands for *t*_1_ incrementation and echo–antiecho selection are inserted here. (5) Pulse and receiver phase cycles are standardized between modules. (6) Comments for PFGs are compatible with the TopSpin *gppp* script. (7) Instructions for generating shaped pulses using TopSpin’s WaveMaker software. (8) Comments describing each parameter appear in the parameter setup screen. (9) Instructions for processing AU programs are encoded here, along with information about the specific modules used and a timestamp that ensures reproducibility.

Using this information, GENESIS then constructs the pulse program in several steps (Figure 3). The pulse program begins with header comments including information about the pulse program and constituent modules. Parameter definitions for each module are then collated, taking particular care to avoid duplicate definitions for parameters used in multiple modules. The main section, which contains the actual instructions for the pulse sequence, is then put together: this is done mostly by concatenating individual modules, with some context-sensitive blocks such as purge pulses, pulsed field gradients (PFGs), or ASAP mixing^18^ placed between modules. Appropriate commands for looping and incrementation of parameters such as phase cycles, *t*_1_ delays, and PFG amplitudes for echo–antiecho selection are added at the end of the main section. Following this, additional comments containing descriptive text for parameters (displayed in TopSpin’s *ased* parameter setup screen), as well as PFG and shaped pulse information (which allow direct setup using the *gppp* and *wvm* commands), are inserted. Finally, we also specify the exact modules used in the pulse program, the GENESIS version number, and a timestamp: this is important for reproducibility purposes.

An immediate problem of directly concatenating pulse program texts from different modules is that a given parameter in one module may take on a different meaning in another module. To avoid such clashes, we have fully standardized all parameters used in the GENESIS pulse programs. These include pulse widths (*p#*), delays (*d#*), constants (*cnst#*), *z*-gradient pulse amplitudes (*gpz#*), and phase cycles (*ph#*), where # represents a nonnegative integer. Where possible, we have chosen meanings that match those in the standard library of Bruker pulse programs, only deviating to avoid otherwise inevitable clashes between different modules. Furthermore, in place of module-specific delays which are often called *DELTA#* in standard library sequences, we have chosen to define new identifiers with more human-readable names inside the pulse program itself. Thus, the delays in an HSQC sequence might be called *DC_HSQC#*, where the first C indicates the indirect-dimension nucleus (^13^C). While this standardization was primarily implemented to facilitate pulse program construction, this also makes it far easier for users to set up NOAH experiments. Since the majority of these parameters are consistent with the standard Bruker library, many of them may be directly read in from the *prosol* relation tables and/or existing parameter sets in TopSpin. Furthermore, since every parameter has the same meaning in every NOAH supersequence, it also makes setting up multiple supersequences an almost trivial task: generally, only the parameters *NBL*, *PULPROG*, and *TD1* need to be changed.

**Figure 4:**
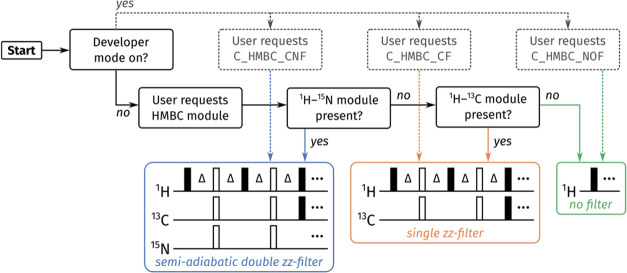
Flowchart illustrating how GENESIS decides the form of the *zz*-filter to be used in a ^1^H–^13^C HMBC module. Dotted lines represent the branch where developer mode is enabled, i.e., the user has full control over which form is used: these are specified using alphabetical labels of the form *C_HMBC_{LABEL}*. Solid lines represent the standard user mode, i.e., developer mode disabled: in this case, GENESIS automatically chooses the appropriate module based on what other modules are present in the supersequence.

Another potential issue is that different versions of a pulse sequence often exist. An example is the *zz*-HMBC module, where the implementation of the *zz*-filter element depends on whether ^13^C-bound and/or ^15^N-bound ^1^H magnetization needs to be retained for later modules.^17,19^ Since the user only specifies that they want an HMBC module and not the exact details of the *zz*-filter, the GENESIS code must in effect make this choice for the user behind the scenes, in an adaptive fashion that changes depending on what other modules the user selects (Figure 4). Advanced users may, however, circumvent this and make their own choice by entering developer mode, where each HMBC version is assigned a unique label *C_HMBC_{LABEL}* (these labels are explained in more detail on the website). The sensitivity-enhanced HSQC^25,26^ (seHSQC, abbreviated “S^+^”/Sp) module presents a similar case. If the seHSQC module is followed by one or more homonuclear modules such as a COSY or NOESY, then the ZIP element^22,23^ is automatically inserted at the beginning of the seHSQC module, to preserve the bulk magnetization required by the later modules. However, if the seHSQC module is placed at the end of the sequence, then the ZIP element is omitted to maximize sensitivity.

To maximize flexibility within a sequence, the GENESIS pulse programs make substantial use of acquisition flags, a feature within TopSpin that allows for conditional compilation of pulse program segments. By defining one or more “symbols” inside the *zgoptns* TopSpin parameter, users may choose whether to include additional features in the pulse program, such as multiplicity editing in HSQC sequences, zero-quantum suppression,^27^ and solvent suppression. The benefit of this is that there is no need for users to store multiple different pulse sequences which differ only in small, isolated segments.

As a final point for this section, we touch on the issue of reproducibility, which is a key consideration for scientific code such as GENESIS.^28^ Although one of the primary aims of GENESIS is to release new updates to NOAH supersequences in a timely fashion, it is also important that old releases of pulse sequences remain available, so that scientific results using these pulse sequences may be reproduced. Furthermore, each release is accompanied by a suite of processing scripts; these may also be modified over time, and to ensure compatibility with the pulse sequences, old versions of the scripts must also be kept available.

To ensure that NMR experiments run with GENESIS pulse programs and scripts are always reproducible, each pulse program and script is marked with a version number (labeled (9) in Figure 3). Old versions of GENESIS may be obtained using the following formula: to access version vX.Y.Z (where X, Y, Z are integers), navigate to the URL https://nmr-genesis.co.uk/X/Y/Z. For example, the release version that accompanies this paper is labeled v2.1.0; this can be accessed at https://nmr-genesis.co.uk/2/1/0. While earlier versions are also available and functional, these should be treated as “prerelease” versions, to be used at the reader’s own risk. As an alternative, the GENESIS code can be obtained from GitHub at https://github.com/yongrenjie/genesis and run entirely offline, allowing users to rewind to any desired version. Instructions on how to use this are provided in the repository description.

In its current form, GENESIS is not capable of creating arbitrary pulse sequences; its scope is limited to NOAH supersequences. However, this remains a viable target for the future: instead of combining NOAH modules to form a supersequence, one may instead consider combining pulse sequence elements (e.g., spin echoes, INEPT, zero-quantum suppression, decoupling, or solvent suppression schemes) to form a pulse sequence. At an even more granular level, individual building blocks (e.g., pulses, delays, PFGs) could be strung together to construct a pulse sequence in an interactive fashion.

## New NOAH Improvements in Genesis

We now detail a few recent improvements to NOAH supersequences, all of which are already implemented in the live GENESIS web page. It is worth emphasizing that the modular nature of GENESIS means that the addition of a new module enables the creation of all possible supersequences containing that module (of which there may be hundreds); traditionally, only around five of these might have been included with a publication. Likewise, simply changing the underlying code for any existing NOAH module is sufficient to immediately propagate changes to every relevant supersequence.

### ^13^C HMBC Module

**Figure 5:**
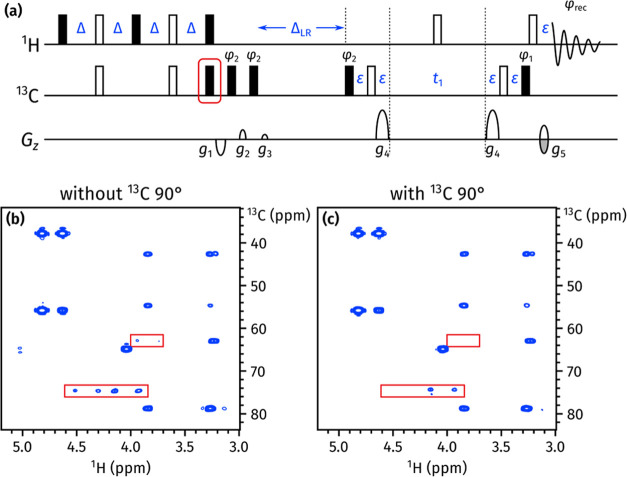
(a) NOAH *zz*-HMBC pulse sequence, with the newly added ^13^C 90° pulse outlined in red. The delays are Δ = 1 / (4 · ^1^*J*_CH_) and Δ_LR_ = 1 / (2 · ^*n*^*J*_CH_); ε is the minimum time required for a PFG plus the subsequent recovery delay. Phase cycling is performed as follows: φ_1_ = *x*, −*x*; φ_2_ = *x*, *x*, −*x*, −*x*; and φ_rec_ = *x*, −*x*, −*x*, *x*. All PFGs have duration 1 ms; amplitudes as a fraction of the maximum *z*-gradient pulse strength (55.7 G/cm) are as follows: *g*_1_ = −15%; *g*_2_ = 10%; *g*_3_ = 5%; *g*_4_ = 80%; and *g*_5_ = ±40.2%. (b) HMBC spectrum obtained using the original *zz*-HMBC module, i.e., without the added 90° pulse. ^1^*J*_CH_ artifacts are highlighted in red boxes. (c) HMBC spectrum obtained with the added 90° pulse. Spectra were obtained on a 700 MHz Bruker AV III equipped with a TCI H/C/N cryoprobe; the sample used was 40 mM andrographolide in DMSO-*d*_6_.

The *zz*-HMBC module is ordinarily placed first in a supersequence because the *zz*-filter element allows magnetization of protons directly coupled to ^13^C (and/or ^15^N) to be preserved for later modules.^17,19^ Specifically, the *zz*-filter acts as a 90° excitation pulse on ^12^C-bound protons while leaving ^13^C-bound protons along +*z*. This is largely accomplished in practice, as evidenced by the fact that the intensities in subsequent HSQC-type modules are barely perturbed. However, due to instrumental imperfections and/or J-coupling mismatch, not all of the ^13^C-bound ^1^H magnetization is perfectly retained: in particular, the *zz*-filter also generates a degree of antiphase magnetization of the form H_*x*_C_*z*_. This antiphase magnetization is later refocused during the low-pass J-filter (LPJF) to give in-phase magnetization, eventually ending up as one-bond correlation artifacts in the HMBC spectrum (Figure 5b).

A simple solution to this is to add a ^13^C 90° pulse at the end of the *zz*-filter (Figure 5a): this converts any antiphase magnetization to double- or zero-quantum magnetization, which is subsequently dephased by the LPJF. This idea has previously been used by Luy and co-workers in CLIP-HSQC experiments to remove antiphase contributions prior to FID detection.^29^ In the event, this small modification proved to have a large impact, almost completely suppressing the one-bond artifacts (Figure 5c). Further comparisons of artifact intensity are provided in Section S2 of the Supporting Information.

### ^15^N HMQC Module

**Figure 6:**
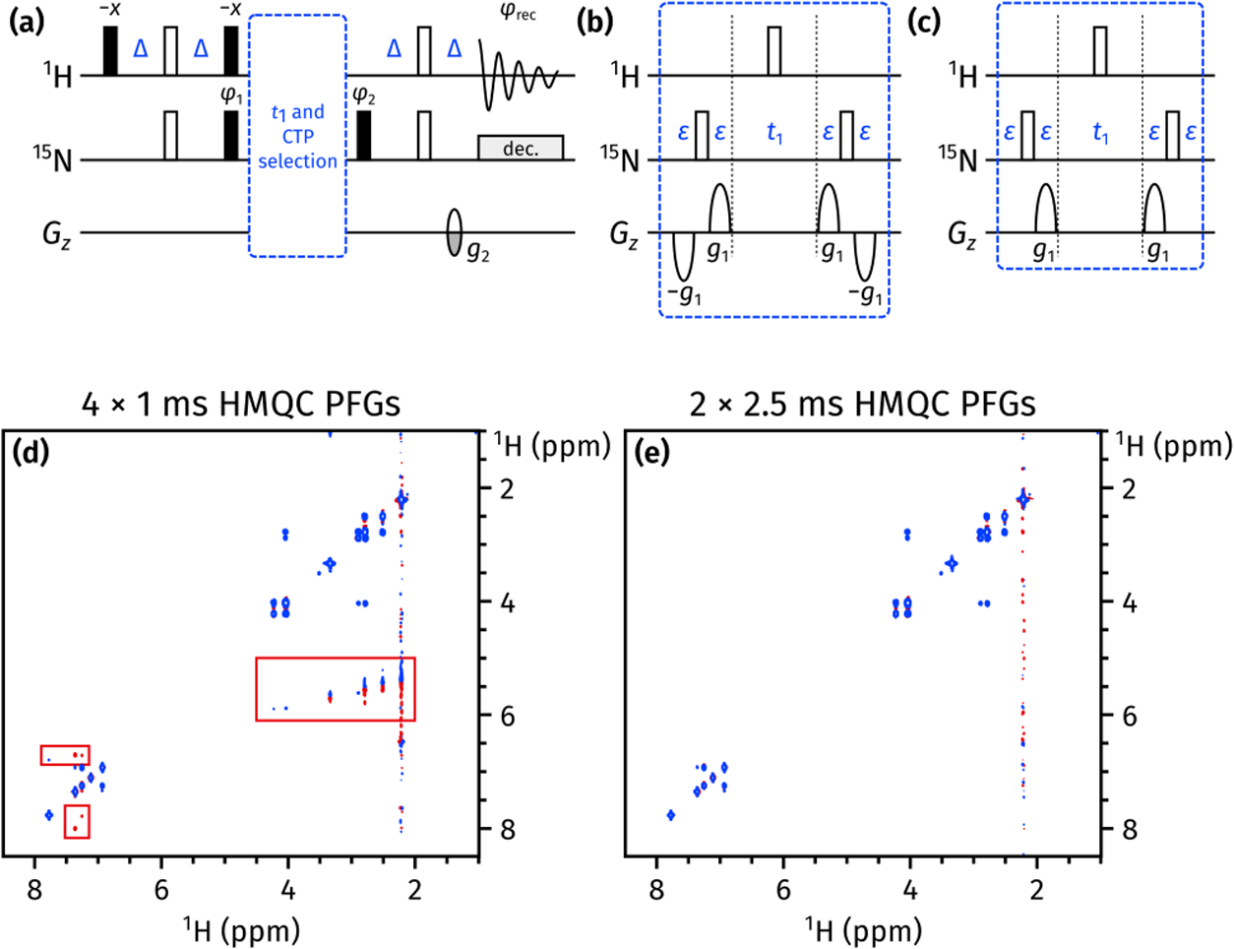
(a) General outline of the NOAH ^15^N HMQC module. *g*_2_ has a duration which matches that of *g*_1_ (explained in the text), and an amplitude of ±*n* · 8.1%, where *n* is the number of PFGs bracketing the *t*_1_ period. *g*_1_ has an amplitude of 80% in all cases. All other symbols have the same meaning as in Figure 5. (b) Previously published CTP selection scheme for the HMQC module, with four PFGs each of duration 1 ms. (c) New CTP selection scheme for the HMQC module, with two PFGs each of duration 2.5 ms. (d,e) CLIP-COSY spectra obtained from a NOAH-3 MS^+^C^c^ supersequence (^15^N HMQC + ^13^C seHSQC + CLIP-COSY), using the HMQC selection schemes shown in (b) and (c), respectively. The wing artifacts in the former spectrum are highlighted in red boxes. Spectra were obtained on a 700 MHz Bruker AV III equipped with a TCI H/C/N cryoprobe; the sample used was 50 mM zolmitriptan in DMSO-*d*_6_.

We have recently described the occurrence of “wing artifacts” in homonuclear modules, which arise from bulk magnetization which evolves in one of the two halves of *t*_1_ in a preceding *heteronuclear* module, such as an HMQC (Figure 6a).^23^ These artifacts can be removed in an elegant manner by ensuring that each half of *t*_1_ in the heteronuclear module contains coherence transfer pathway (CTP) PFGs of equal sign and magnitude, which makes sure that any bulk magnetization undergoing net evolution during *t*_1_ is dephased. At the same time, it is also important that the final refocusing PFG in the heteronuclear module (*g*_2_) has as large an amplitude as possible since it is responsible for dephasing bulk magnetization that is not returned to +*z* just prior to the detection period. The previous ^15^N HMQC module placed pairs of bipolar PFGs (*g*_1_) in both halves of *t*_1_, a scheme that allows *g*_2_ to have an amplitude of 4*g*_1_γ_N_/γ_H_ (Figure 6b). Although this leads to excellent artifact suppression in the ^15^N HMQC itself, wing artifacts are apparent in downstream modules (Figure 6d) because the opposing PFGs cancel each other out and do not enforce any CTP selection on the bulk magnetization.

To suppress these “wing artifacts” in later modules, it proves better to only use two PFGs during *t*_1_ (one in either half) and to lengthen their duration such that the final PFG *g*_2_ provides sufficient CTP selection in the HMQC module itself (Figure 6c). This strategy was previously described for the ^15^N seHSQC module;^23^ here, we have also applied it to the HMQC module with success (Figure 6e). This change causes no significant difference in the sensitivity of the resulting spectra (Figure S3).

### Pure Shift and 2D J Modules

In previous work,^23^ we described how ^1^H–^15^N modules could be implemented with optional “*k*-scaling.”^30^ This entails a reduction in the number of *t*_1_ increments (by a factor of *k*), in return for a corresponding increase in the number of transients per increment, with no overall change in the experiment time. In particular, for the HMQC experiment, this allowed modest gains in sensitivity as *J*_HH_ splittings were no longer resolved in the indirect dimension.

A simple extension of this protocol to homonuclear ^1^H–^1^H modules enables experiments such as 2D J-resolved or pseudo-2D pure shift spectroscopy to be incorporated into NOAH supersequences. In both cases, the number of *t*_1_ increments needed (16–32) is far smaller than the typical number required for a 2D experiment (128–256). In particular, at present, we have implemented a family of PSYCHE experiments, namely: the original pseudo-2D PSYCHE (abbreviated “P”); the triple spin-echo (TSE)-PSYCHE experiment (“P^T^”), which provides improved robustness toward strong coupling; and the PSYCHE 2D J experiment (“J”), which yields pure absorption-mode line shapes.^31−33^ On top of this, there is also a magnitude-mode 2D J module available (“J^qf^”).

**Figure 7:**
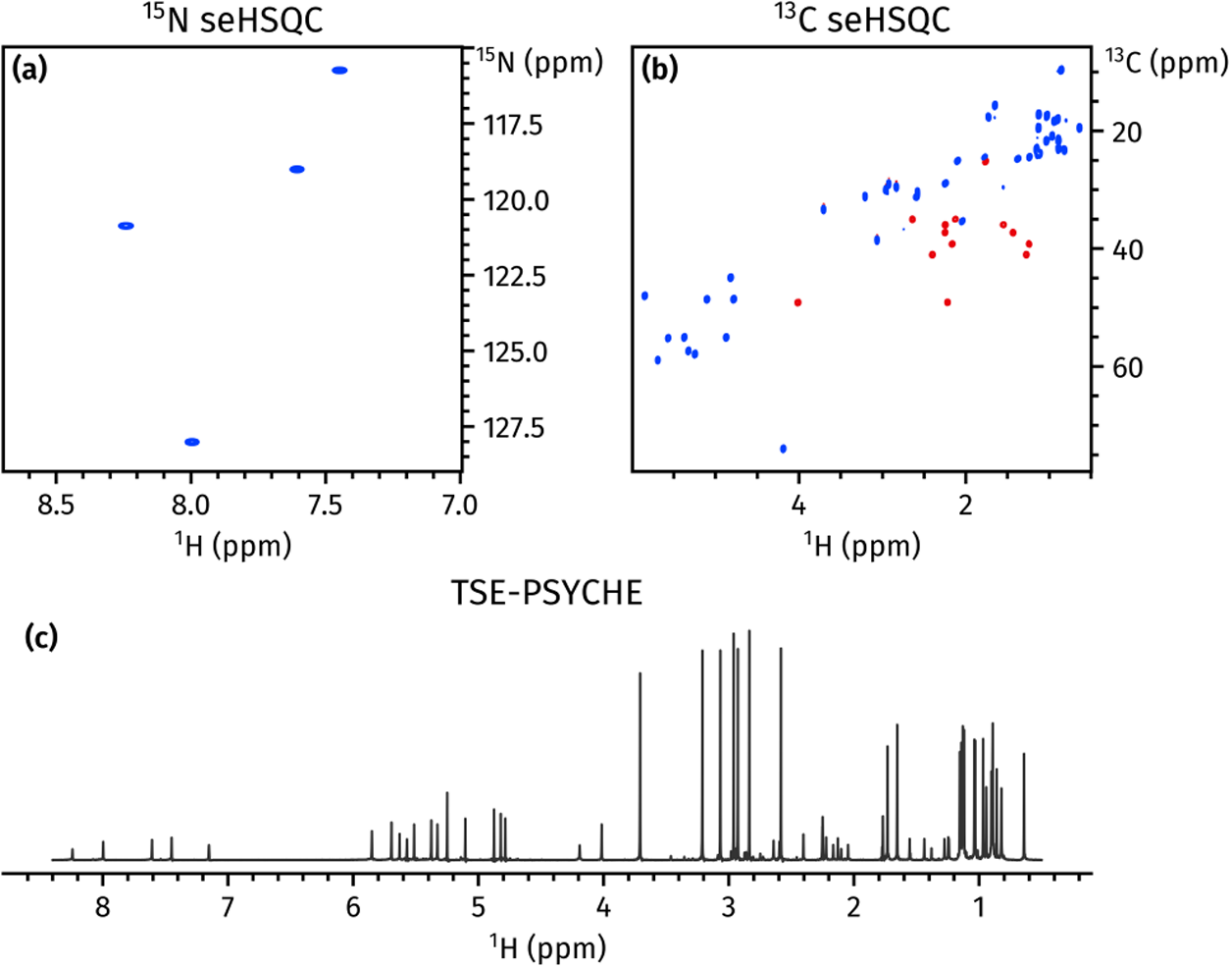
Spectra obtained from a NOAH-3 S_N_^+^S^+^P^T^ supersequence. (a) ^15^N sensitivity-enhanced HSQC^23^ (256 *t*_1_ increments, 2 scans per increment). (b) ^13^C sensitivity-enhanced HSQC^22,23^ (256 *t*_1_ increments, 2 scans per increment). (c) 1D TSE-PSYCHE pure shift spectrum^31^ (saltire flip angle of 15°, 32 chunks, 16 scans per chunk, 8-step SAPPHIRE averaging). Spectra were obtained on a 700 MHz Bruker AV III equipped with a TCI H/C/N cryoprobe; the sample used was 50 mM cyclosporin A in C_6_D_6_.

In PSYCHE spectra, the flip angle of the chirp or saltire pulses used in the J-refocusing element provides the experimentalist with a choice: a larger flip angle provides greater sensitivity but at the cost of increased artifacts.^33^ One advantage of acquiring PSYCHE spectra in NOAH supersequences is that the increased number of transients compensates for the sensitivity losses inherent to PSYCHE and other pure shift techniques. Thus, the user can choose a smaller flip angle (10–15°) to maximize spectral purity, without incurring the usual drawback of increased experiment time. On top of that, extra transients may also be used to carry out SAPPHIRE averaging to suppress artifacts arising from J-modulation;^34^ this feature is enabled by default in GENESIS. An example of a NOAH-3 supersequence with the TSE-PSYCHE module is shown in Figure 7.

### Solvent Suppression

The addition of solvent suppression to NOAH supersequences is more involved than for a typical NMR experiment because the water signal must be adequately suppressed in all modules, ideally without affecting any other magnetization components. The HMQC- and HSQC-type NOAH modules in fact provide good intrinsic solvent suppression because the magnetizations of all ^1^H spins not coupled to heteronuclei—including that of water—are returned to +*z* at the end of the sequence. However, other modules require the addition of specific solvent suppression techniques.

Two options are currently available, namely, presaturation (during the recovery delay *d*_1_ and the mixing time in NOESY modules) and excitation sculpting placed just prior to acquisition in homonuclear (^1^H–^1^H) modules.^35^ The refocusing element used in the latter is a combination of a shaped and hard 180° pulse. Both presaturation and excitation sculpting can be independently turned on or off using the -*DPRESAT* and -*DES zgoptns* acquisition flags in TopSpin, respectively.

### *splitx_au* Processing

NOAH data processing is done using the *splitx_au* AU program; this is responsible for creating separate data sets containing the data for each module, defining any required processing parameters, and processing each data set using module-specific AU programs (e.g., *noah_hsqc* for ^13^C HSQC data). Previously, the names of module-specific AU programs had to be manually specified as the *USERP#* series of processing parameters. In contrast, with GENESIS pulse programs, this information is directly embedded within the pulse program itself; furthermore, we have modified the *splitx_au* AU program to obtain the requisite list of AU programs by parsing the pulse program text. Users therefore no longer need to provide the *USERP#* parameters, which makes setting up multiple different supersequences a much smoother process. If necessary, it is possible to override these preselected AU programs by explicitly specifying the *USERP#* parameters, allowing for customized processing.

### Nonuniform Sampling (NUS)

With the GENESIS pulse programs, we also introduce a new and more user-friendly implementation of NUS. NOAH experiments do not work “out of the box” with TopSpin’s conventional NUS setup routine: some special adjustments must be made by manually generating the list of increments to be sampled and adjusting the *t*_1_ delays accordingly within the pulse sequence looping. Previously, this was accomplished using a Python script that created a new pulse program for each supersequence, e.g., *noah3_BSC.nus*.^18^ We have modified this approach such that NUS is instead controlled by a *zgoptns* acquisition flag *-DNUS*, with the benefit that the same pulse program can be used for both uniform and nonuniform sampling. Although a (different) Python script is still required for initialization, this means that it is no longer necessary to keep two separate instances of the same pulse sequence in TopSpin, thus simplifying the usage of NUS in NOAH supersequences.

## Conclusions

In this article, we have demonstrated how the modular nature of NOAH supersequences can be exploited in the systematic generation of pulse programs containing any arbitrary set of constituent modules. This provides a real, practical way to obtain a myriad of possible supersequences which have been hitherto inaccessible. Users can create customized sequences which are tailored to their needs and also access newly implemented or improved modules immediately upon release. Here, we have described several such enhancements, including built-in solvent suppression flags, new pure shift and 2D J modules, the reduction of artifacts in HMBC and HMQC modules, and improved processing routines. All of these, as well as any future updates, are and will be available via the GENESIS website (https://nmr-genesis.co.uk).

## Supporting Information Available

Detailed analysis of NOAH combinations and comparisons of pulse sequences (PDF)
