# Supplementary material for: Modular Pulse Program Generation for NMR Supersequences
Source: Anal Chem. 2022 Jan 20;94(4):2271–8. doi: 10.1021/acs.analchem.1c04964 (PMC9082496; doi:10.1021/acs.analchem.1c04964)
Supplement: Supplementary file 1 — ac1c04964_si_001.pdf [file ac1c04964_si_001.pdf]

Supporting Information

for

Modular Pulse Program Generation for NMR Supersequences

Jonathan R. J. Yong,[1] Ēriks Kupče,[2] Tim D. W. Claridge[1,*]

[1] *Chemistry Research Laboratory, Department of Chemistry, University of Oxford, Mansfield Road, Oxford, OX1 3TA, United Kingdom*

[2] *Bruker UK Ltd, R&D, Coventry CV4 9GH, United Kingdom*

* `tim.claridge@chem.ox.ac.uk`

Contents

S1 Number of NOAH combinations **S2**

S2 More HMBC artifact comparisons **S3**

S3 HMQC sensitivity comparison **S5**

S4 Raw data and figures **S6**

S1 Number of NOAH combinations

In this section we count the total number of “viable” NOAH combinations, available from the normal user mode of the GENESIS website, using the inclusion–exclusion principle. As of version 2.1.0 of the website, there are five categories of modules:

- HMBC (2 choices, including “none”)
- ^{15}N–^{1}H (3 choices, including “none”)
- ^{13}C–^{1}H #1 (5 choices, including “none”)
- ^{13}C–^{1}H #2 (9 choices, including “none”)
- ^{1}H–^{1}H (19 choices, including “none”)

To a first approximation, there are therefore $2 \cdot 3 \cdot 5 \cdot 9 \cdot 19 = \mathbf{5130}$ combinations. Since we included the “none” options in this product, this figure includes “lesser” supersequences such as NOAH-4 and lower. However, this does contain some invalid combinations, namely:

1. All five “none” modules selected (**1**).
2. “NOAH-1” combinations with only one module: $1 + 2 + 4 + 8 + 18 = \mathbf{33}$. These are technically valid experiments (and the pulse programs generated by the website *will* in fact function correctly), but they are no different from standard 2D experiments so the NOAH description does not truly apply to these.
3. “NOAH-6” combinations where there is one experiment in each of the first four categories, and a “double” experiment (e.g., COSY + NOESY) in the last category. There are 6 such modules, for a total of $1 \cdot 2 \cdot 4 \cdot 8 \cdot 6 = \mathbf{384}$ combinations. These are perfectly sound from a scientific perspective, but cannot be executed in current versions of TopSpin as the parameter `NBL` (number of modules) has a maximum value of 5.
4. NOAH-2 or NOAH-3 combinations consisting of the HMBC module directly followed by a ^{1}H–^{1}H homonuclear module: **18**. These can be run, but are likely to produce lower-quality homonuclear spectra as the HMBC module dephases bulk magnetization.
5. Duplicate, identical combinations where the same ^{13}C–^{1}H module (e.g., HSQC) was selected either from the third or the fourth column. There are 4 such modules, so we must subtract $2 \cdot 3 \cdot 4 \cdot 19 = \mathbf{456}$ combinations.
    - Note, however, that some of these duplicate combinations were *already* rejected in step (2) above. Thus, we need to add back **4** “NOAH-1” combinations.

This yields a total number of $5130 - 1 - 33 - 384 - 18 - 456 + 4 = \mathbf{4242}$ possible NOAH supersequences. Note that, unlike the original paper,[1] this does not take into account options that are set using acquisition flags, such as multiplicity editing or solvent suppression. This also does not include modules that are only available via the “developer mode” interface.

S2 More HMBC artifact comparisons

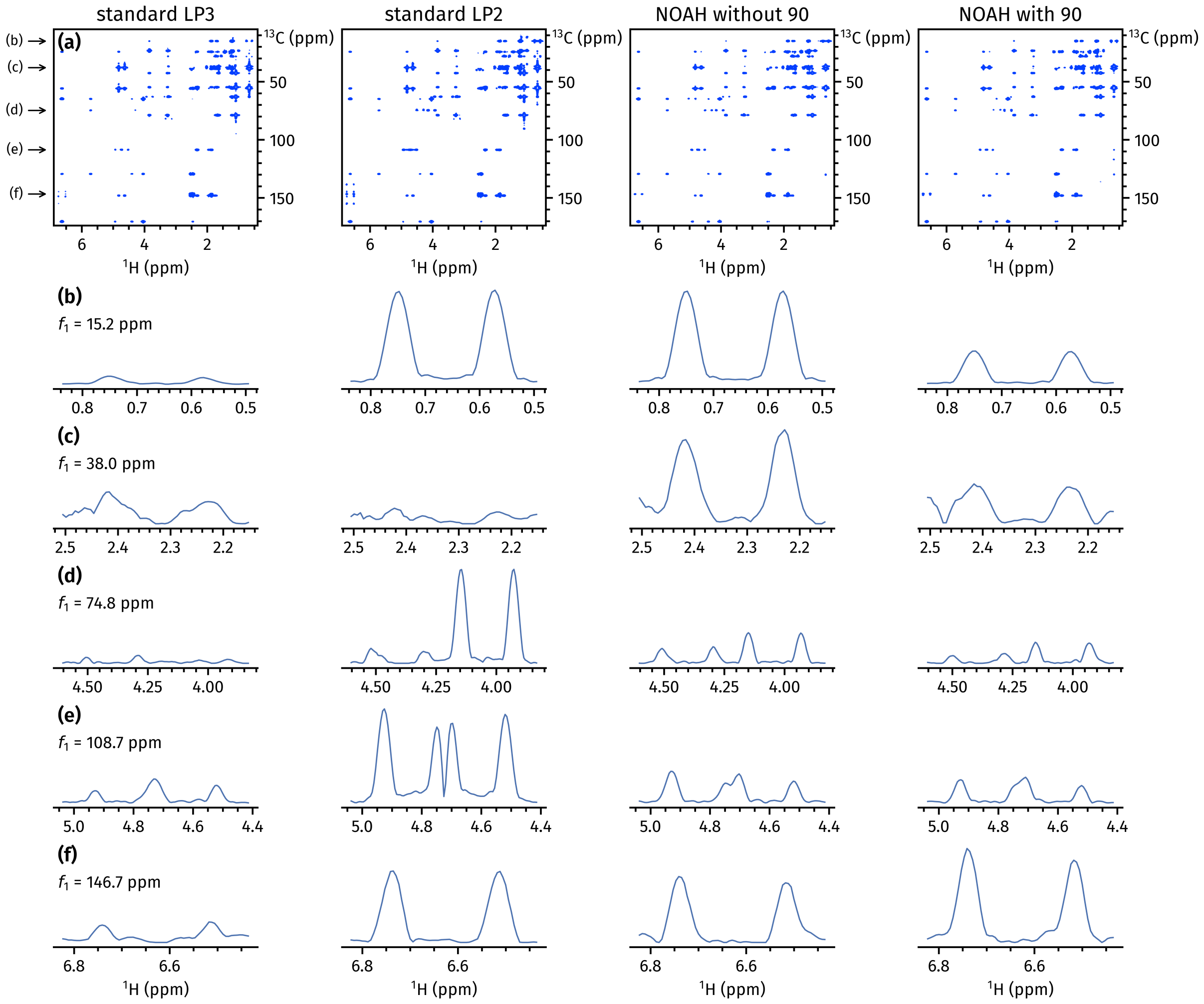

Figure S1: Comparisons of HMBC spectra obtained using the Bruker standard library sequences[2] `hmbcetgpl3nd` (with a third-order LPJF, first column) and `hmbcetgpl2nd` (with a second-order LPJF, second column); and the NOAH *zz*-HMBC module (pulse sequence in Figure 5a), before and after the added ^{13}C 90° pulse (third and fourth columns respectively). It should be noted that the NOAH *zz*-HMBC module features a second-order LPJF as well. (a) The full 2D spectra, plotted with the same contour levels. (b)–(f) Multiple f_1 traces through the 2D spectra showing the $^1J_{CH}$ artifacts. The spectra within each row are plotted with the same *y*-axis range. In all cases except (f), the addition of the 90° pulse leads to far better artifact suppression; in many cases, its performance is comparable to the standard library third-order LPJF. Spectra were obtained on a 700 MHz Bruker AV III equipped with a TCI H/C/N cryoprobe; the sample used was 40 mM andrographolide in DMSO-d_6.

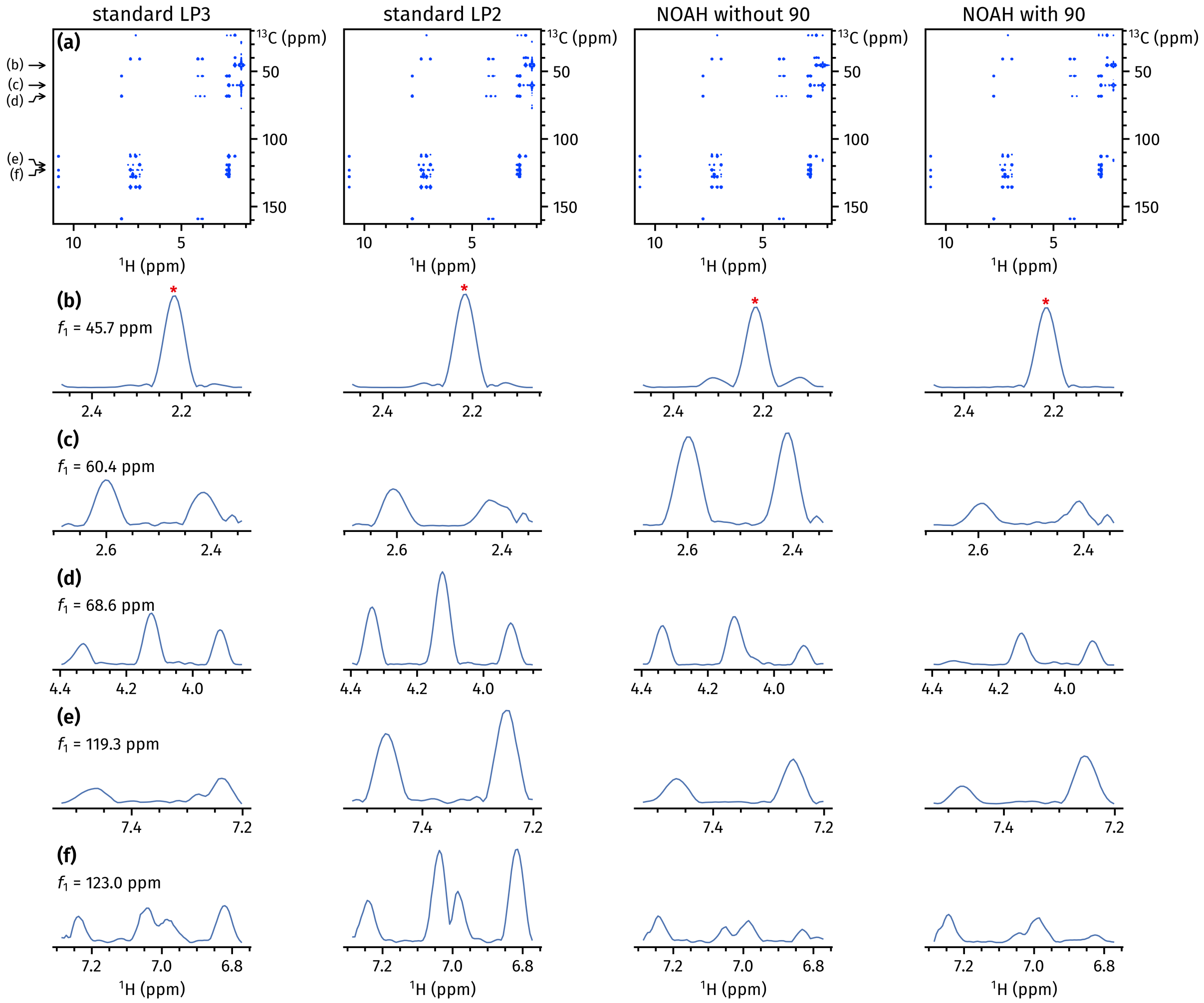

Figure S2: The same as in Figure S1, but instead acquired with a sample of 50 mM zolmitriptan in DMSO-d_6. Note that the peak labelled with an asterisk in (b) is a genuine correlation. It is, however, flanked by a pair of $^1J_{CH}$ artifacts (most visible in the third column, i.e., NOAH *zz*-HMBC without added 90° pulse).

S3 HMQC sensitivity comparison

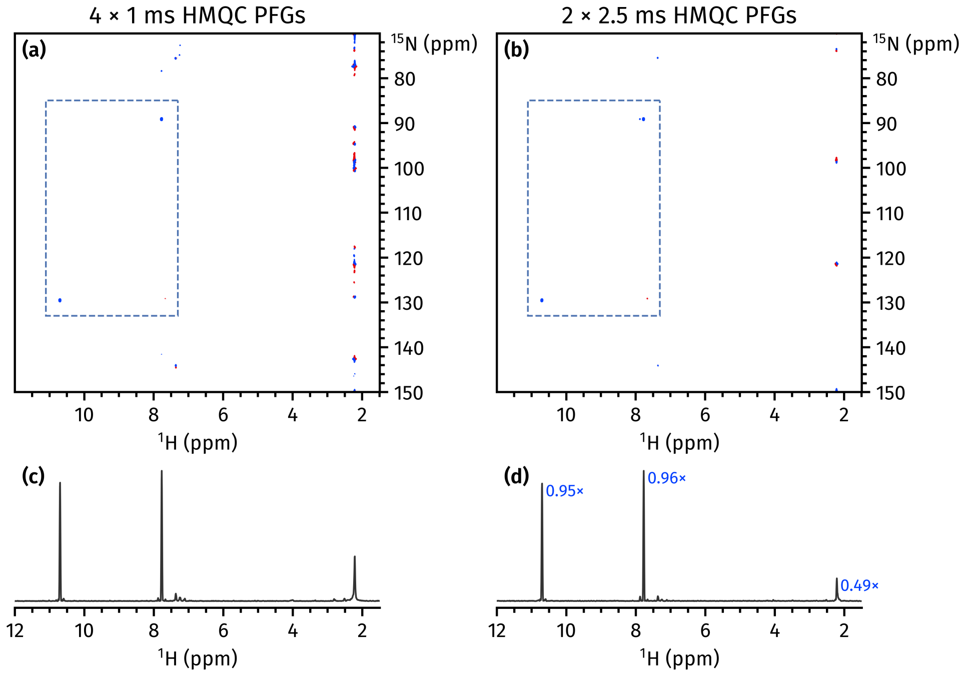

Figure S3: Comparisons of ^{1}H–^{15}N HMQC spectra obtained using the two CTP selection schemes shown in Figure 6 of the main text. (**a**)–(**b**) Full 2D spectra. The two desired signals are boxed. Note the artifacts at $f_2 = 2.2$ ppm; these arise from bulk magnetization that is not fully dephased by the PFGs in the HMQC module.[3] These artifacts are also present in the standard Bruker HMQC / seHSQC sequences, and have comparable intensity to the unmodified NOAH spectrum in (a). (**c**)–(**d**) Positive projections of the spectra onto the f_2 axis. The relative intensities of the peaks and artifacts are shown in (d). Switching to the improved CTP selection scheme leads to no significant change in the intensity of the desired peaks (≥ 95%), and halves the artifact intensity. We previously reported similar results with the ^{1}H–^{15}N seHSQC module.[3] Spectra were obtained on a 700 MHz Bruker AV III equipped with a TCI H/C/N cryoprobe; the sample used was 50 mM zolmitriptan in DMSO-d_6.

S4 Raw data and figures

The scripts used to generate the figures in this paper are available on GitHub at https://github.com/yongrenjie/genesis-paper. The raw NMR datasets are required to generate these figures. Due to their size, they are not uploaded in the same repository: they may be separately downloaded, either from a GitHub release (https://github.com/yongrenjie/genesis-paper/releases/tag/final-revision), or from Zenodo (DOI: 10.5281/zenodo.5822959).

References

(1) Kupče, Ē.; Claridge, T. D. W. NOAH: NMR Supersequences for Small Molecule Analysis and Structure Elucidation. *Angew. Chem., Int. Ed.* **2017**, *56*, 11779–11783, DOI: 10.1002/anie.201705506.

(2) Cicero, D. O.; Barbato, G.; Bazzo, R. Sensitivity Enhancement of a Two-Dimensional Experiment for the Measurement of Heteronuclear Long-Range Coupling Constants, by a New Scheme of Coherence Selection by Gradients. *J. Magn. Reson.* **2001**, *148*, 209–213, DOI: 10.1006/jmre.2000.2234.

(3) Yong, J. R. J.; Hansen, A. L.; Kupče, Ē.; Claridge, T. D. W. Increasing sensitivity and versatility in NMR supersequences with new HSQC-based modules. *J. Magn. Reson.* **2021**, *329*, 107027, DOI: 10.1016/j.jmr.2021.107027.
